# The Accuracy of Digital Face Scans Obtained from 3D Scanners: An In Vitro Study

**DOI:** 10.3390/ijerph16245061

**Published:** 2019-12-12

**Authors:** Pokpong Amornvit, Sasiwimol Sanohkan

**Affiliations:** Department of Prosthetic Dentistry, Faculty of Dentistry, Prince of Songkla University, Hat Yai, Songkhla 90110, Thailand; pokpong_am@yahoo.com

**Keywords:** digital, face scanners, accuracy, three-dimensional analysis, face analysis, facial driven design

## Abstract

Face scanners promise wide applications in medicine and dentistry, including facial recognition, capturing facial emotions, facial cosmetic planning and surgery, and maxillofacial rehabilitation. Higher accuracy improves the quality of the data recorded from the face scanner, which ultimately, will improve the outcome. Although there are various face scanners available on the market, there is no evidence of a suitable face scanner for practical applications. The aim of this in vitro study was to analyze the face scans obtained from four scanners; EinScan Pro (EP), EinScan Pro 2X Plus (EP+) (Shining 3D Tech. Co., Ltd. Hangzhou, China), iPhone X (IPX) (Apple Store, Cupertino, CA, USA), and Planmeca ProMax 3D Mid (PM) (Planmeca USA, Inc. IL, USA), and to compare scans obtained from various scanners with the control (measured from Vernier caliper). This should help to identify the appropriate scanner for face scanning. A master face model was created and printed from polylactic acid using the resolution of 200 microns on x, y, and z axes and designed in Rhinoceros 3D modeling software (Rhino, Robert McNeel and Associates for Windows, Washington DC, USA). The face models were 3D scanned with four scanners, five times, according to the manufacturer’s recommendations; EinScan Pro (Shining 3D Tech. Co., Ltd. Hangzhou, China), EinScan Pro 2X Plus (Shining 3D Tech. Co., Ltd. Hangzhou, China) using Shining Software, iPhone X (Apple Store, Cupertino, CA, USA) using Bellus3D Face Application (Bellus3D, version 1.6.2, Bellus3D, Inc. Campbell, CA, USA), and Planmeca ProMax 3D Mid (PM) (Planmeca USA, Inc. IL, USA). Scan data files were saved as stereolithography (STL) files for the measurements. From the STL files, digital face models are created in the computer using Rhinoceros 3D modeling software (Rhino, Robert McNeel and Associates for Windows, Washington DC, USA). Various measurements were measured five times from the reference points in three axes (x, y, and z) using a digital Vernier caliper (VC) (Mitutoyo 150 mm Digital Caliper, Mitutoyo Co., Kanagawa, Japan), and the mean was calculated, which was used as the control. Measurements were measured on the digital face models of EP, EP+, IPX, and PM using Rhinoceros 3D modeling software (Rhino, Robert McNeel and Associates for Windows, Washington DC, USA). The descriptive statistics were done from SPSS version 20 (IBM Company, Chicago, USA). One-way ANOVA with post hoc using Scheffe was done to analyze the differences between the control and the scans (EP, EP+, IPX, and PM). The significance level was set at *p* = 0.05. EP+ showed the highest accuracy. EP showed medium accuracy and some lesser accuracy (accurate until 10 mm of length), but IPX and PM showed the least accuracy. EP+ showed accuracy in measuring the 2 mm of depth (diameter 6 mm). All other scanners (EP, IPX, and PM) showed less accuracy in measuring depth. Finally, the accuracy of an optical scan is dependent on the technology used by each scanner. It is recommended to use EP+ for face scanning.

## 1. Introduction

The human face not only shows the physical anatomical landmarks of a person’s identity, but reveals the psychological make-up or personalization [1]. Facial morphology and analysis are important for various disciplines, such as craniofacial-maxillofacial surgery, orthodontics, prosthodontics, pedodontics, biometrics, and forensic odontologists [2,3,4]. The conventional methods of facial analysis include two-dimensional (2D) photographic, Vernier caliper, and bevel protractor measurements, to measure 2D projection distances and angles [2,5,6]. Recently, there has been a digital dental era due to the massive advancement and evolution in optical scanning and designing technology that has led a shift from 2D to three-dimensional (3D) technology, with the use of 3D leading to disruption in the treatment modality [7,8,9]. The facial landmarks can be recorded digitally from the 3D face scanning using the scanner and can be used for facial recognition, capturing facial emotions, facial cosmetic planning and surgery, maxillofacial rehabilitation, etc. [10,11,12,13,14]. The computer aided design/computer aided manufacturing (CAD/CAM) system, milling systems, rapid prototyping, three-dimensional (3D) scanning, and 3D printing have revolutionized and created new modalities in medicine and dentistry, which improved the speed and accuracy of treatment [15,16,17]. Currently, CAD/CAM is widely used in dentistry for the fabrication of inlays, onlays, veneers, crowns, implant prosthesis, and full-mouth reconstruction [16,18,19]. In addition, in dentistry, 3D imaging using dental cone beam computed tomography (CBCT) offers volumetric data on jaw bones and teeth, which helps in presurgical diagnosis, preoperative planning, and transferring a preoperative plan for actual treatment outcome for oral rehabilitation [20,21,22]. Similarly, augmented reality is also an emerging technology in medicine and dentistry, and this includes an accurate display of either static or dynamic diagnostic images via the use of a visor or specific glasses, which is useful, especially in implant surgery [23].

For aesthetic and full mouth rehabilitation, although the semi-adjustable articulator and face bow are commonly used, they are based on the average values and cannot be individualized unless the fully adjustable articulator is used [24]. However, due to high technical skills requirements and errors from the users, the measurements may have questionable accuracy. Hence, the 3D face scan and intraoral scan can be integrated with a CBCT scan for the facial analysis, occlusion analysis, and even digital/virtual face bow transfer and full mouth rehabilitation [22,25,26,27,28,29,30,31,32]. The data from CBCT, intraoral scans, and facial scans can be superimposed to create a 3D “virtual patient” for better diagnosis, treatment planning, and patient outcomes [22]. Mangano et al. [32] found that the combination of intraoral and face scans allowed them to successfully restore fully edentulous patients with maxillary overdentures supported by four implants and a CAD/CAM PEEK bar. The facially driven design is a technique which considers the facial esthetics, facial profile, proportions, and harmony for the oral rehabilitation, and uses digital smile design to make aesthetically pleasing faces. This approach is important for aesthetic dental restorations considering hard and soft tissues, which results in enhanced smiling, self-esteem, and self-confidence of patients [1,33,34,35,36]. Higher accuracy improves the quality of the data recorded from the face scanner, which ultimately improve the outcome [37]. Although there are various face scanners available in the market, there is no evidence of a suitable scanner for practical applications.

Hence, the aim of this in vitro study was to analyze the face scans obtained from four scanners:—EinScan Pro (EP), EinScan Pro 2X Plus (EP+) (Shining 3D Tech. Co., Ltd. Hangzhou, China), iPhone X (IPX) (Apple Store, Cupertino, CA, USA), and Planmeca ProMax 3D Mid (PM) (Planmeca USA, Inc. IL, USA)—and to compare scans obtained from various scanners with the control (measured from Vernier caliper). This helped to identify the appropriate scanner for face scanning.

## 2. Materials and Methods

**Figure 1:**
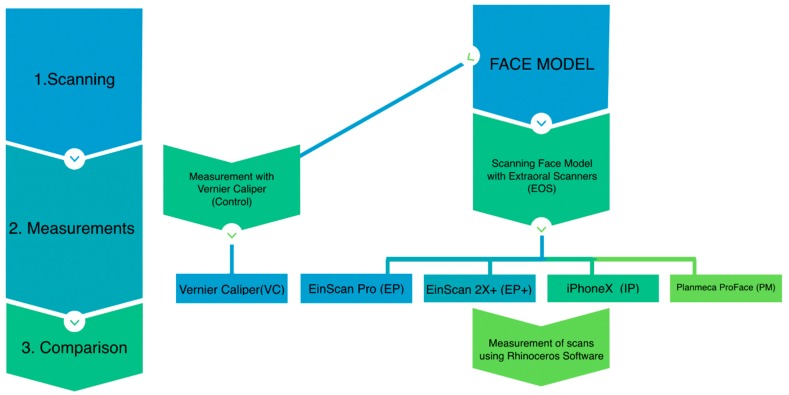
Study overview. The study involved scanning, measurements, and comparison.

The overview of the study is shown in Figure 1 and the method can be divided into three parts: scanning, measurements, and comparison.

### 2.1. Scanning

**Figure 2:**
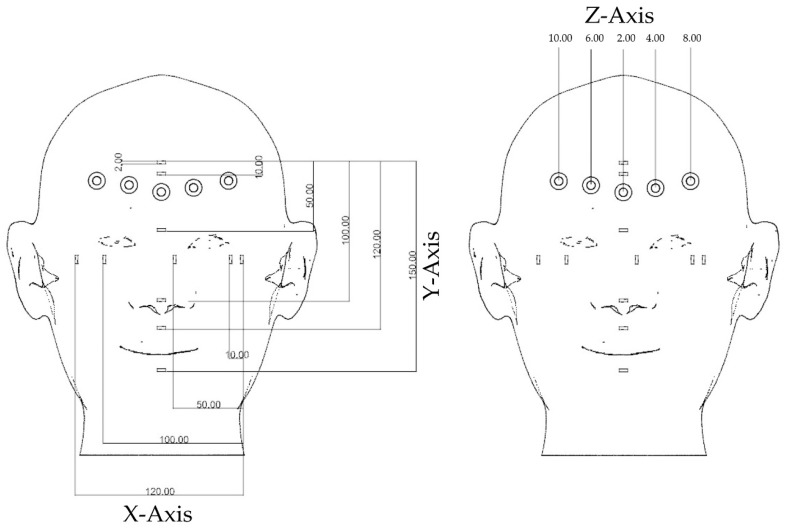
Reference points marked on the model and various measurements. X-axis (length), y-axis (length), and z-axis (depth).

**Figure 3:**
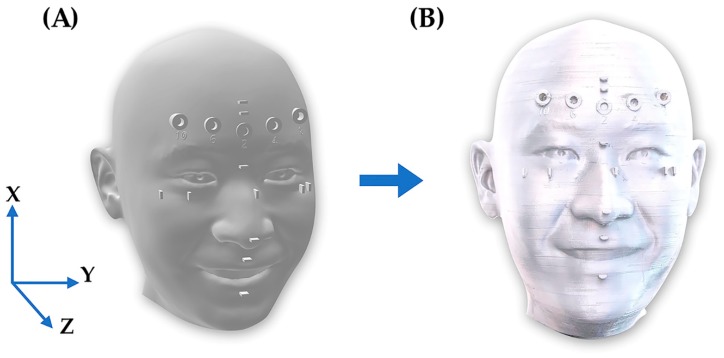
3D face model rendering (**A**) and 3D printed master face model (**B**).

At first, a face model was designed in Rhinoceros 3D modeling software (Rhino, Robert McNeel and Associates for Windows, Washington DC, USA) with shape, size, and ratios close to the human face. Reference points marked on the model and various measurements along the x axis (length), y axis (length), and z axis (depth) are shown in Figure 2. The face model was printed from polylactic acid using the resolution of 200 microns on x, y, and z axes (Figure 3).

**Figure 4:**
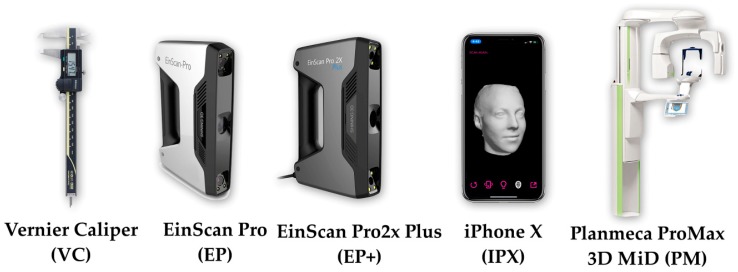
Vernice caliper and various face scanners used in this study. EinScan, EinScan Pro 2X Plus, iPhone X, and Planmeca ProMax 3D Mid.

Then, the master face model was 3D scanned with four scanners five times according to the manufacturer’s recommendations: EinScan Pro (Shining 3D Tech. Co., Ltd. Hangzhou, China), EinScan Pro 2X Plus (Shining 3D Tech. Co., Ltd. Hangzhou, China) using Shining Software, iPhone X (Apple Store, Cupertino, CA, USA) using the Bellus3D Face Application (Bellus3D, version 1.6.2, Bellus3D, Inc. Campbell, CA, USA), and Planmeca ProMax 3D Mid (PM) (Planmeca USA, Inc. IL, USA) (Figure 4). Scan data files were saved as stereolithography (STL) files for the measurements. From the STL files, digital face models were created in the computer using Rhinoceros 3D modeling software (Rhino, Robert McNeel and Associates for Windows, Washington DC, USA).

### 2.2. Measurement

Various measurements were measured five times from the reference points on three axes (x, y, and z) using a digital Vernier caliper (VC) (Mitutoyo 150 mm Digital Caliper, Mitutoyo Co., Kanagawa, Japan), and the mean was calculated, which is shown in Figure 2. The same measurements as measured before were measured on the digital face models of EP, EP+, IPX, and PM using Rhinoceros 3D modeling software (Rhino, Robert McNeel and Associates for Windows, Washington DC, USA).

### 2.3. Comparison

The measurements of the various scanners were compared with the Vernier caliper. The descriptive statistics were done from SPSS version 20 (IBM Company, Chicago, USA). One-way ANOVA with post hoc using Scheffe was done to analyze the difference between the control and the scans (EP, EP+, IPX, and PM). The significance level was set at *p* = 0.05.

## 3. Results

### 3.1. Scanning Time

Table 1 shows the scanning time, processing time, and total time for the scanning process of four scanners (EP, EP+, IPX, and PM). The scanning process consists of scanning and data processing. Scanning time is the time taken for scanning the face. Data processing includes data editing, generating points, meshing, removing artifacts, making a solid model, and finishing the face model. The fastest scan was done by IPX with Bellus3D (0.57 ± 0.03 min), and was followed by PM (0.7 ± 0.05 min), EP (6.77 ± 0.15 min), and finally, EP+ (9.4 ± 0.21 min).

### 3.2. Scanning Accuracy

The best scanning accuracy was shown by EP+, which was followed by EP, IPX, and finally, PM (Figure 3). EP showed that medium scanning accuracy and the least accuracy was shown by IPX and PM. Regarding the depth, EP+ showed accuracy of 2 mm in depth (diameter 6 mm). All other scanners (EP, IPX, and PM) showed less scanning accuracy when measuring depth.

Table 2 shows the mean measurements along the x-axis of the face model from the Vernier caliper (VC) and various scans (EP, EP+, IPX, and PM), and the comparisons of various scans from the VC.

For the EP, the X3, X4, and X5 measurements showed significant differences (*p* < 0.01) compared to VC. For the EP+, all the measurements on the z-axis showed no significant difference to the VC. For the IPX, the X4 measurement showed a significant difference (*p* < 0.01) compared to the VC. The X1 measurement could not be measured because scanning the point for the measurement of X1 could not be captured in the IPX scan. Hence, from the results in x-axis, it can be implied that the EP showed inaccuracy in capturing the length of more than 50 mm. However, EP+ showed accuracy in recording the length until 120 mm. IPX showed accuracy in capturing the length from 10 mm until 50 mm, but failed to record the details and had difficulties while measuring length (X1). Similarly, Table 3 shows the mean measurements in the y-axis of face model from the Vernier caliper (VC) and various scans (EP, EP+, IPX, and PM), and the comparisons of various scans from the VC.

For the EP, it showed that the Y3, Y4, Y5, and Y6 measurements showed significant differences (*p* < 0.01) compared to the VC. For the EP+, all the measurements showed no significant difference to the VC. For the IPX, Y6 showed significant difference (*p* < 0.01) compared to the VC. For the PM, Y1, Y3, Y5, and Y6 showed significant difference (*p* < 0.01) compared to the VC. The X1, Y1, and Y2 measurements could not be measured because scanning the point for the measurement of Y1 could not be captured in the IPX scan. Similarly, from the results in y-axis, the EP showed inaccuracy in capturing the length more than 50 mm (similar to X-axis). In addition, EP+ showed accuracy in recording the length until 150 mm. IPX showed accuracy in capturing the length from 50 mm until 120 mm, but failed to record the details and had difficulties while measuring length (Y1 and Y2).

Finally, Table 4 shows the mean measurements in z-axis of face model from the caliper (VC) and various scans (EP, EP+, IPX, and PM) and the comparisons of scans from the VC.

For both the EP and EP+, it showed that all the measurements in z-axis showed significant differences (*p* < 0.01) compared to the VC. For IPX, all the measurements in z-axis could not be measured because scanning the point for the measurement of Z-axis could not be captured in the IPX scan. Hence, all the scanners were unable to record clearly, the depth of more than 2–4 mm with a diameter of 6 mm. The IPX showed the least accuracy in recording the depth of more than 2 mm.

**Figure 5:**
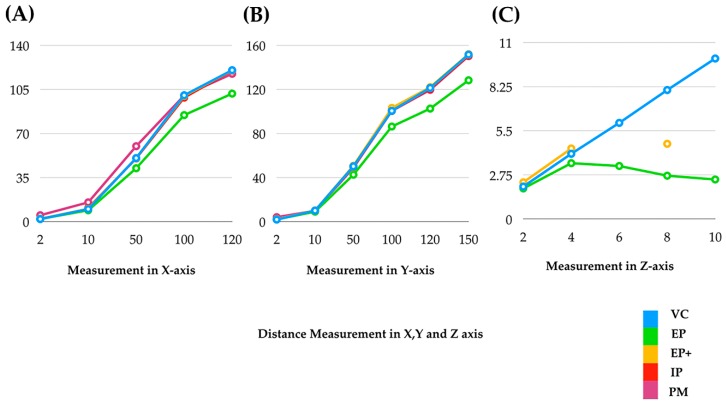
Mean measurements of caliper and three scanners in three axes: (**A**) x-axis (length), (**B**) y-axis (length), and (**C**) z-axis (depth). Discontinuity in a line shows the inability in measurements.

**Figure 6:**
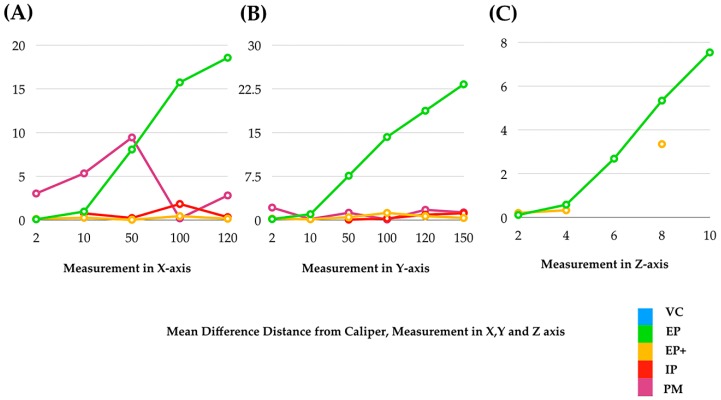
Mean difference of various measurements of various scanners from the caliper in three axes: (**A**) x-axis (length), (**B**) y-axis (length), and (**C**) z-axis (depth). Discontinuity in a line shows the inability in measurements.

Figure 5 shows the mean measurements along the x-axis (length), y-axis (length), and z-axis (depth) of VC, and three scanners. Discontinuity in a line shows the inability in measurements. Figure 6 shows the mean differences of various measurements in the x-axis (length), y-axis (length), and z-axis (depth) of three scanners from the caliper. It showed that the mean difference of the measurements increased as the distance increased (from 2 to 120 mm in the x-axis, from 2 to 150 mm in the y-axis, and from 4 to 10 mm in the z-axis). The factors affecting the accuracy in the z-axis, or depth, are light intensity, focus, distance of objects from the scanner, and deviation of light.

## 4. Discussion

Facial and dental measurements analyses help in smile design and esthetic rehabilitation [38,39,40,41,42]. With the huge advancements in digital technology, it has been widely applied in medicine and dentistry for diagnoses, therapeutics, artificial intelligence, and augmented reality [43]. The face scanning and digital impression-making of teeth are widely used now a days [44]. The advantages of scanners are simplified clinical procedure; time efficiency; patient comfort; no use of plaster/dental stone, as a digital model is prepared in the computer; better patient communication and motivation; simplifying clinical procedures for both the dentist and the laboratory technician; ease of communication with technician; and ease of fabrication of prosthesis [19,44,45,46,47]. 

Accuracy is key in all clinical applications in prosthesis and scanners should be able to detect an accurate impression [48,49,50]. In this study, a face model was fabricated using the fused deposition modeling technique and this study analyzed the details and accuracy of face scans obtained from four scanners (EP, EP+, IPX, and PM) and compared the scans obtained from various scanners with the control (measured from Vernier caliper). This helped to identify the appropriate scanner for face scanning. In this study, EP+ showed the highest accuracy (accuracy until 150 mm of length). EP showed medium scanning accuracy (accurate until 10 mm of length). IPX and PM were the least accurate (accuracy from 10 mm to 120 mm in length). For IPX, various measurements (X1, Y1, and Y2) could not be measured because they could not be captured in the scan, as IPX and PM showed. In addition, IPX showed accuracy in capturing the length from 10 mm until 50 mm in the x-axis and from 50 mm until 120 mm in the y-axis, with failure to record the details and difficulty while measuring. This might be due the ability of the video capturing capacity of the IPX. The inaccuracy of PM scanner may be due scanning from a short distance due to the lesser field of vision. Our study is similar to the study done by Zhao et al. [2], wherein they compared the practical accuracy of optical facial scanners for facial deformity patients using a high-accuracy industrial “line-laser” scanner (Faro), stereophotography (edMD), and a “structured light” facial scanner (FaceScan). The respective 3D accuracy of stereophotography and structured light facial scanners obtained for facial deformities were 0.58 ± 0.11 mm and 0.57 ± 0.07 mm. The 3D accuracies of different facial partitions were inconsistent; the measurements at the middle face had the showed the highest accuracy. Although the accuracies of two facial scanners were lower than their nominal accuracies, they all met the requirements for oral clinic use. However, from the results of our study, only EP+ is suitable for face scanning.

Mangano et al. [48] compared the accuracy of five different intraoral scanners (IOSs) in the impressions of single and multiple implants. They used plaster models representative of a partially edentulous maxilla to be restored with a single crown and a partial prosthesis, and a totally edentulous maxilla to be restored with a full-arch. They concluded that the IOSs showed significant differences between them, both in trueness and in precision. The mathematical error increased in the transition from single crown to partial prosthesis up to full-arch, both in trueness and in precision. A similar study was done by Braian et al. [51], wherein they studied the trueness and precision under repeatable conditions for different IOSs when scanning fully edentulous arches with multiple implants, and they found low precision when scanning fully edentulous arches with multiple implants. This result is similar to our study: as the scanning length increased, the accuracy decreased. They mentioned that the accuracy can be enhanced by reducing the span of scanning, and ensuring the scanned surfaces exhibit minimal irregularities [49]. Furthermore, Braian et al. [52] studied the accuracies of IOSs for scanning dentate and edentulous casts and they found that the significant differences were found in scanning edentulous and dentate scans for short arches and complete arches. Trueness for complete-arch scans was <193 μm (edentulous scans), and was <150 μm for dentate scans. Trueness for short-arch scans was <103 μm (edentulous scans) and <56 μm for dentate scans.

Similarly, for the depth measurement, all the optical scanners showed less accuracy; i.e., were unable to record clearly, a depth of more than 2 mm for diameter 6 mm. The IPX showed the least accuracy in recording the depth of more than 2 mm. Inaccuracy in recording the depth might be due to failure of passing the light into the depth while scanning. For the depth measurement in z-axis, none of the scanners were able to capture it accurately. In this study, a hole of 2 mm was used, which was too narrow to pass the light to the bottom of the hole. The scanners used in this study (EP, EP+, IPX, and PM) are optical scanners. The light from the scanner’s projects in various patterns to the surface and records the 3D picture. The software analyzes and creates the 3D model. The EP projects the visible line pattern, the EP+ projects a QR code-like pattern, the IP projects one QR code-like with infrared rays, and the PM projects X-rays. Light or optical scanning shows it is also acceptable but may have distortion from the light intermittent. IPX uses infrared for face scanning using the Bellus3D Face application to capture hard and soft tissue. In another study, it was found that there was no error in recording the tissue of depth of 5 mm for diameter 4.5 mm by a TRIOS scanner Model S1P (3Shape Trios A/S, Copenhagen, Denmark) for the fabrication of polyether ether ketone (PEEK) abutment for the implant retained finger prosthesis [53].

Van der Meer et al. [54] studied the accuracy of the IOSs by scanning high precision PEEK cylinders and they found the errors and it increased in distance and/or angulations in arch due to an accumulation of registration errors of the patched 3D surfaces. The registration errors may vary in magnitude depending on the scanning technology and the registration algorithms [55]. This all can be eliminated with advancement in the technology. In addition, the scanners may be difficult when scanning shiny, reflective, or transparent objects. The calibration is also done to compensate the errors that have occurred during scanning.

In addition, the fastest scan was done by IPX (0.57 min). PM: 0.7 min; EP, 6.7 min; and EP+, 9.4 min. The faster scanning of IPX and PM might be due to lower accuracy of the scanning and resolution of the scanned file. It was found that there was no significant difference between EP and EP+, which might be due to similar accuracy of the scanning and resolution of the scanned file. In this study, the descending order of the rendering of the 3D scan files was EP+, EP, IPX, PM. In the clinical situation, if the capture details are required, such as the teeth surfaces, auditory canal, or nostril, more accuracy of the scanner or technique of the scan is required. In addition, in a procedure such as digital face bow recording, accuracy is important for accurate recording of an occlusion to the face. IOS can be combined with a facial scanner for oral rehabilitation purposes. Mangano et al. [32] combined intraoral and face scanning for the CAD/CAM fabrication of implant-supported bars for maxillary overdentures. Hence, the scanners which were used in our study can be combined with the IOSs for the esthetic oral rehabiliation. The results from this study can be implemented in capturing landmarks, as shown in Table 5. The capturing difficulty of the facial structures can be classified as follows.

Many factors influence the accuracy of the 3D scanner, such as ability to record details, accuracy, scanning principles, span of scanning, size of scanning area, arch length, surface irregularities, temperature, relative humidity, and illumination [56,57]. The 3D scanning uses one of the various scanning technologies—laser triangulation, structured light, photogrammetry, contact-based, and laser pulse (time of flight or lidar) [58]. Contact-based is the best for the surface scanning but it depends on the probe size. The disadvantages of scanners include costs for the machines, difficulty in handing, technique-sensitive difficulties when capturing the deeper tissues, and rendering [45,58,59,60]. The future of digital scanning is expected to involve wide availability of scanners at lower costs with high quality and accuracy for various dental and medical applications.

## 5. Conclusions

The following conclusions can be drawn from this study:

- The accuracy of a 3D scanner is affected by the scanning length and pattern of scanning.
- The accuracy of an optical scan is dependent on the technology used by each scanner.
- Among the scanners evaluated, EinScan Pro 2X Plus (EP+) showed the highest accuracy (accuracy until 150 mm of length). EinScan Pro (EP) showed moderate accuracy (accurate until 10 mm of length). iPhone (IPX) and ProMax 3D Mid (PM) showed the least accuracy (accuracy from 10 mm to 120 mm in length).
- In addition, EP+ showed accuracy measuring the 2 mm of depth (diameter 6 mm). All other scanners (EP, IPX, and PM) showed less accuracy measuring depth.
- Hence, it is recommended to use EinScan Pro 2X Plus for the face scan for facial driven design and other scanning purposes.
- For measuring the depth of more than 2 mm, these scanners are not recommended. Further development of the scanners is needed for accurately measuring depth.

## Figures and Tables

**Table 1:** Scanning and processing times of various scans (EinScan Pro (EP), EinScan Pro 2X Plus (EP+), iPhone X (IPX), and Planmeca ProMax 3D Mid (PM)).

Scanners	Scanning Time (min)	Data Processing Time (min)	Total Time for Scanning Process (min)
EP	2.14 ± 0.03	4.63 ± 0.11	6.77 ± 0.15
EP+	2.12 ± 0.01	7.28 ± 0.2	9.4 ± 0.21
IPX	0.29 ± 0.01	0.28 ± 0.04	0.57 ± 0.03
PM	0.22 ± 0.01	0.48 ± 0.05	0.7 ± 0.05

**Table 2:** Measurements in x-axis of the face model from the Vernier caliper (VC) and various scans (EP, EP+, IPX, and PM), and the comparisons of various scans from the VC.

Measurements	Groups	Mean ± SD (mm)	Comparison from Vernier Caliper (VC) Using One-Way ANOVA			
			VC vs. EP	VC vs. EP+	VC vs. IPX	VC vs. PM
X1	VC	2.11 ± 0.04	0.82	0.62	NA	<0.001 *
	EP	2.2 ± 0.15				
	EP+	2.25 ± 0.37				
	IPX	NA				
	PM	5.15 ± 1.04				
X2	VC	10.02 ± 0.05	0.21	0.94	0.39	<0.001 *
	EP	9.04 ± 0.44				
	EP+	10.29 ± 0.25				
	IPX	10.8 ± 1.18				
	PM	15.36 ± 2.15				
X3	VC	50.48 ± 0.25	<0.001 *	1	0.96	<0.001 *
	EP	42.41 ± 0.51				
	EP+	50.51 ± 0.4				
	IPX	50.23 ± 1.36				
	PM	59.92 ± 3.34				
X4	VC	100.28 ± 0.06	<0.001 *	0.79	<0.01 *	0.99
	EP	84.55 ± 0.51				
	EP+	99.82 ± 0.63				
	IPX	98.43 ± 1.11				
	PM	100.08 ± 0.94				
X5	VC	120.18 ± 0.05	<0.001 *	0.97	0.77	0.064
	EP	116.3 ± 0.25				
	EP+	120.02 ± 0.127				
	IPX	119.83 ± 0.99				
	PM	117.36 ± 2.88				

SD = standard deviation; NA = not available. * Significant at *p* < 0.05.

**Table 3:** Measurements in the y-axis of the face model from the Vernier caliper (VC) and various scans (EP, EP+, IPX, and PM), and the comparisons of various scans from the VC.

Measurements	Groups	Mean ± SD (mm)	Comparison from Vernier Caliper (VC) Using One-Way ANOVA			
			VC vs. EP	VC vs. EP+	VC vs. IPX	VC vs. PM
Y1	VC	1.97 ± 0.15	0.48	0.25	NA	<0.001 *
	EP	2.15 ± 0.09				
	EP+	2.22 ± 0.389				
	IPX	NA				
	PM	4.1 ± 0.46				
Y2	VC	10.08 ± 0.05	0.04 *	0.82	NA	0.966
	EP	9.06 ± 0.43				
	EP+	10.24 ± 0.48				
	IPX	NA				
	PM	9.90 ± 0.90				
Y3	VC	50.21 ± 0.05	<0.001 *	0.62	0.99	0.027 *
	EP	42.60 ± 0.24				
	EP+	50.70 ± 0.81				
	IPX	50.13 ± 0.8				
	PM	48.93 ± 1.49				
Y4	VC	100.52 ± 0.67	<0.001 *	0.14	0.95	1
	EP	86.28 ± 0.22				
	EP+	101.76 ± 0.96				
	IPX	100.81 ± 0.99				
	PM	100.41 ± 0.57				
Y5	VC	121.30 ± 0.18	<0.001 *	0.4	1.65	0.029 *
	EP	102.56 ± 0.27				
	EP+	121.98 ± 0.71				
	IPX	120.37 ± 0.93				
	PM	119.53 ± 1.18				
Y6	VC	151.49 ± 0.11	<0.001 *	0.62	<0.001 *	0.028 *
	EP	128.22 ± 0.54				
	EP+	151.86 ± 0.32				
	IPX	150.30 ± 0.60				
	PM	150.15 ± 0.92				

SD = standard deviation; NA = not available. * Significant at *p* < 0.05.

**Table 4:** Measurements in z-axis of the face model from the Vernier caliper (VC) and various scans (EP, EP+, IPX, and PM), and the comparisons of scans from the VC.

Measurements	Groups	Mean ± SD (mm)	Comparison from Vernier Caliper (VC) Using One-Way ANOVA			
			VC vs. EP	VC vs. EP+	VC vs. IPX	VC vs. PM
Z1	VC	2.02 ± 0.08	0.03 *	<0.001 *	NA	NA
	EP	1.9 ± 0.06				
	EP+	2.23 ± 0.18				
	IPX and PM	NA				
Z2	VC	4.06 ± 0.03	<0.001 *	<0.001 *	NA	NA
	EP	3.48 ± 0.09				
	EP+	4.39 ± 0.13				
	IPX and PM	NA				
Z3	VC	5.99 ± 0.05	<0.001 *	<0.001 *	NA	NA
	EP	3.3 ± 1.48				
	EP+	1.85 ± 0.07				
	IPX and PM	NA				
Z4	VC	8.04 ± 0.02	<0.001 *	<0.001 *	NA	NA
	EP	2.7 ± 0.44				
	EP+	4.69 ± 0.19				
	IPX and PM	NA				
Z5	VC	10 ± 0.05	<0.001 *	<0.001 *	NA	NA
	EP	2.46 ± 0.05				
	EP+	1.95 ± 0.05				
	IPX and PM	NA				

SD = standard deviation; NA = not available. * Significant at *p* < 0.05.

**Table 5:** The classification of the capturing difficulty of the facial structures.

Capturing Difficultness	Landmarks
Easy	Forehead, cheek, and chin
Medium	Ear lobe and eye lids
Hard	Teeth, extra auditory canal, and nostril
